# Molecular Characteristics of Catalytic Nitrogen Removal from Coal Tar Pitch over γ-Alumina-Supported NiMo and CoMo Catalysts

**DOI:** 10.3390/ijms241411793

**Published:** 2023-07-22

**Authors:** Kyoung-Hwan Choi, Dong-Jin Seo, Yu-Jin Kim, San-Seong Cho, Yu-Jin Han, Inchan Yang, Chel-Woo Kim, Kyeongseok Oh, Jung-Chul An, Joo-Il Park

**Affiliations:** 1 Department of Biochemical Engineering, Seoul University, 28, Yongmasan-ro 90-gil, Jungnang-gu, Seoul 02192, Republic of Korea; 2 Department of Chemical & Biological Engineering, Hanbat National University, Daejeon 34158, Republic of Korea; 3 Korea Institute of Energy Research, 50 UNIST-gil, Eonyang-eup, Ulju-gun, Ulsan 44919, Republic of Korea; 4 Carbon Materials Research Group, Research Institute of Industrial Science & Technology (RIST), Pohang 37673, Republic of Korea; 5 Department of Chemical and Biological Engineering, Inha Technical College, Incheon 22212, Republic of Korea

**Keywords:** coal tar pitch, denitrogenation, hydrotreatment, FT-ICR MS

## Abstract

The removal of nitrogen from coal tar pitch (CTP) through the hydrodenitrogenation (HDN) of CTP and its molecular behavior were evaluated in the presence of NiMo/γ-alumina and CoMo/γ-alumina catalysts. Fourier transform ion cyclotron resonance mass spectrometry with atmospheric pressure photoionization was used to analyze the complicated chemical classes and species of CTP and the treated products at the molecular level. Nitrogen species were qualitatively analyzed before and after hydrotreatment. A single-stage hydrotreatment with an HDN catalyst resulted in a high sulfur removal performance (85.6–94.7%) but a low nitrogen removal performance (26.8–29.2%). Based on relative abundance analyses of nitrogen and binary nitrogen species, C*_c_*H*_h_*-N*_n_*S*_s_* was the most challenging species to remove during HDN treatment. Furthermore, prior hydrodesulfurization was combined with HDN treatment, and the dual hydrotreatments yielded a significantly improved nitrogen removal performance (46.4–48.7%).

## 1. Introduction

Needle coke is produced by heating petroleum or coal tar pitch (CTP) to high temperatures, causing the material to form elongated needlelike structures [1].

Needle coke produced using petroleum pitch is typically of a higher quality than that produced using CTP because of the higher purity and lower nitrogen content of petroleum pitch [2], which is a by-product of petroleum refining. Petroleum pitch is obtained from the residue of crude oil distillation, whereas CTP, which is a by-product of coal processing, is obtained by distilling coal tar (CT) [3].

The selection of needle coke produced using petroleum pitch or CTP depends on several criteria, including the quality, purity, availability, and cost of the pitch and the desired properties of the needle coke. Therefore, CTP remains of interest because of its low cost and commercial-scale availability [4].

CT, as a source of CTP, is a by-product of coal processing and contains various contaminants, including nitrogen-containing compounds such as pyridine, quinoline, and indole [5]. The removal of nitrogen from CT or CTP is essential in reducing their environmental effects and preventing puffing in various industrial applications.

Several methods of nitrogen removal from CT have been developed, including biological [6], chemical [7], and physical processes [8]. Biological processes [9], such as microbial degradation and bioremediation, are often slow and require specific conditions. Chemical processes include acid washing [10,11] and oxidation [12]. Acid washing involves treating CT with an acid to remove nitrogen-containing compounds; however, this process is ineffective in removing all types of nitrogen-containing compounds. Oxidation methods, such as ozonation and the Fenton method, effectively remove nitrogen from CT [13]. However, these methods are expensive and require the use of hazardous chemicals. Physical methods include adsorption and membrane filtration. Adsorption involves the use of activated carbon or other adsorbents [14,15,16], whereas membrane filtration involves the use of membranes [17,18]. These methods are effective in removing nitrogen-containing compounds but may not be economical.

As CT or CTP is a heavy residue produced during coal processing, a catalytic removal method, such as hydrodenitrogenation (HDN), is a promising approach for removing nitrogen. This is because the catalytic removal of nitrogen from CTP exhibits several advantages over other nitrogen removal methods, including a high efficiency, a low cost, and the capacity to proceed under mild reaction conditions. However, the selection of the catalyst and reaction conditions depends on the nitrogen-containing compounds within CTP and the desired level of nitrogen removal. Further research is required to optimize the catalytic removal of nitrogen from CTP and develop highly efficient and cost-effective catalysts.

In this regard, sufficient molecular data are necessary to resolve the current issue of upgrading CTP to value-added carbon materials, such as premium needle coke. The molecular data of heavy residues in the petroleum/oil industries are obtained using high-pressure liquid chromatography [19], gas chromatography (GC)-time-of-flight mass spectrometry [20], GC with atomic emission detection [21], and GC- or gel permeation chromatography (GPC)-inductively coupled plasma mass spectrometry (ICP-MS) [22]. Although these methods provide similar results to those obtained using conventional analytical methods, such as atomic absorption spectroscopy, elemental analysis, and ICP-MS, they remain limited in terms of the molecular-level compositions of heavy residues.

Fourier transform ion cyclotron resonance mass spectrometry (FT-ICR MS) with various ionization methods, such as electrospray, electron and laser desorption ionization, field desorption/ionization, and atmospheric pressure photoionization (APPI), is a highly advanced analytical tool [23]. Numerous FT-ICR MS studies have been conducted to elucidate the chemical composition of crude oil to understand and predict the characteristics of heavy petroleum at the molecular level. However, detailed information regarding the characteristics of the molecular behavior of coal residue and its oil products is currently unavailable [24]. Current research in the area has focused on combining chromatographic methods, such as gas and liquid chromatography, occasionally employing fractionation. Resolving the reaction pathway at the molecular level and revealing the catalytic behavior using FT-ICR MS are significant for further advancements in this field.

This study establishes an advanced methodology for evaluating the catalytic removal of nitrogen species from CTP at the molecular level by analyzing their behaviors during hydrotreatment over commercial γ-alumina-supported NiMo (NMA) and CoMo (CMA) catalysts. This attempt marks the first time in the coal-related industry and engineering chemistry. To yield meaningful results at the molecular level, FT-ICR MS with APPI was used to characterize the C*_c_*H*_h_*, C*_c_*H*_h_*-S*_s_*, C*_c_*H*_h_*-N*_n_*, C*_c_*H*_h_*-O*_o_*, and C*_c_*H*_h_*-binary and -ternary heteroatom classes (herein, subscripts *c*, *h*, *s*, *n*, and *o* are numbers of elements C, H, S, N, and O) in the feed and products of CTP hydrotreatment.

## 2. Results and Discussion

### 2.1. Overall Reactivity

Table 1 lists the measured sulfur and nitrogen contents in the feed and hydrotreated products. The net changes in the total amounts of sulfur and nitrogen in the CTP samples during hydrotreatment were calculated, and the results are summarized in Table 1. The C/H ratio in the hydrotreated CTP decreases gradually during the single and dual stages of operation over the NMA and CMA catalysts. The total change in the C/H ratio during the single-stage reaction is 15.9% over NMA and 14.3% over CMA, indicating that hydrotreatment over CMA exhibits a higher activity than that over NMA. The dual-stage reaction displays a different trend (both catalysts: 14.8). The total sulfur content in CTP also gradually decreases during the single and dual stages of operation over NMA and CMA. However, the conversion (94.7 wt.%) of the total sulfur content in CTP over CMA is significantly higher than that (86.5 wt.%) over NMA, suggesting that the sulfur species are more easily hydrotreated over CMA. Moreover, an ideal removal of sulfur species is observed during the dual-stage reaction, which is attributed to the high selectivity toward sulfur over the second-stage catalysts, after the diluted catalyst (DM5CQ) is used in the first-stage reaction. Significantly, the total nitrogen content in CTP does not decrease sufficiently in each stage. Even the net amount of nitrogen in CTP decreases by up to 40.0% compared to the original amount of nitrogen in the feed. This is because the nitrogen species in CTP are located at the sterically hindered sites of the carbon agglomerates and/or polar nitrogen species occupying the acid/active sites of hydrogenolysis over the catalysts.

### 2.2. Reactivities of the Molecular Classes

A comparison of the results of bulk analysis and FT-ICR MS remains controversial, as shown in the study by Rodgers [25], wherein the results of FT-ICR MS are explained based on the C/H ratios obtained via bulk analysis. Even when several constraints are assumed owing to the preferential ionization (APPI) of highly aromatic/condensed CTP and its heterogeneous aggregation properties, comparing the fractions of CTP in detail in terms of their polarities and C/H molar ratios should be insightful. Notably, FT-ICR MS with APPI is limited in reflecting the complete, precise molecular distribution, as its selective ionization effectiveness for different molecules may vary. However, the selective ionization effectiveness for the same molecules or class of molecules should be identical or similar. This enables the use of the FT-ICR MS spectra to interpret the relative changes in the various classes of molecules in CTP during hydrotreatment. Table 2 and Table 3 list the relative abundance of each molecular class in CTP after the single- and dual-stage reactions, respectively, as the weighted average intensities due to radical actions and protonated species generated by the positive APPI. The net change in each molecular class during hydrotreatment was calculated based on the difference in each molecular class of the feed and products, and the results are also presented in Table 2 and Table 3. After the single-stage reaction, the relative abundances of most classes containing sulfur (C*_c_*H*_h_*-S*_s_*, C*_c_*H*_h_*-O*_o_*S*_s_*, and C*_c_*H*_h_*-N*_n_*O*_o_*S*_s_*) decrease, whereas the relative abundance of the binary-coordinated sulfur–nitrogen class (C*_c_*H*_h_*-N*_n_*S*_s_*) increases. Notably, the relative abundance of the ternary-coordinated sulfur–nitrogen–oxygen class decreases over NMA and CMA during the single-stage reaction. The increase in the amount of C*_c_*H*_h_* may be attributed to an increase in heteroatom-free hydrocarbons due to effective hydrogenolysis. Therefore, the classes with binary-coordinated sulfur–nitrogen heteroatoms are located at the sterically hindered sites, less active than the single-/ternary-coordinated sulfur classes, and challenging to remove from CTP during hydrotreatment. A comparison of the heteroatom classes reveals that the unitary heteroatom classes in CTP are removed in the order of C*_c_*H*_h_*-S*_s_*, C*_c_*H*_h_*-O*_o_*, and C*_c_*H*_h_*-N*_n_*. The binary heteroatom classes in the asphaltenes are removed in the order of C*_c_*H*_h_*-N*_n_*O*_o_*, C*_o_*H*_h_*-O*_o_*S*_s_*, and C*_c_*H*_h_*-N*_n_*S*_s_*, based on the changes in their net amounts during hydrotreatment. These results suggest that the heteroatom removal reactivities of all species in CTP decrease in the order HDS > hydrodeoxygenation (HDO) > HDN.

Additionally, the net amount of the C*_c_*H*_h_*-N*_n_*S*_s_* class in the first step of the dual-stage reaction increases slightly, by approximately 10%. The increase in the C*_c_*H*_h_*-N*_n_*S*_s_* class in the products during the single- or first-stage reaction may be attributed to (1) the significantly lower HDN reactivities of the classes with nitrogen heteroatoms; (2) the conversion of C*_c_*H*_h_*-N*_n_*O*_o_*S*_s_* via HDO to C*_c_*H*_h_*-N*_n_*S*_s_*, as the reactivities of heteroatom species generally decrease in the order HDS > HDO > HDN; and (3) the polycondensation of the C*_c_*H*_h_*-N*_n_*S*_s_* of CTP. Table 3 also indicates the increase in the net amount of C*_c_*H*_h_* in the products after the single- and dual-stage reactions, which may be attributed to the removal of the heteroatoms of the heteroatom-containing classes in CTP. A detailed discussion on the analysis of the data obtained via (APPI) FT-ICR MS is provided in the subsequent section to analyze the changes in the abundances of various molecular classes and their subclasses, with the DBE values and carbon number distributions.

### 2.3. C_c_H_h_ Class

**Figure 1:**
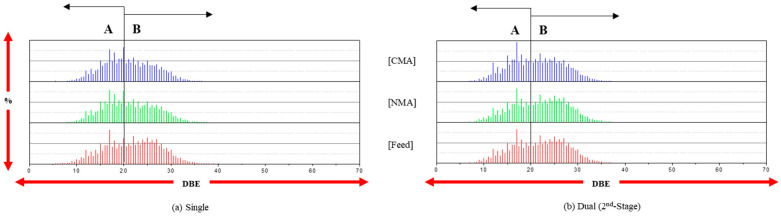
The distribution of DBE (C*_c_*H*_h_* species) at the single and dual stages (only second stage).

The behavior of the *C_c_H_h_* class was characterized via the distribution of the DBE values, as shown in Figure 1. The DBE values of the feed and products (NMA and CMA) are in the range 5–40. Even when the distribution of the DBE values is bimodal (A and B ranges), the B-range abundance (%) is reduced after HDN in the single-stage reaction. However, each HDN (second-stage) after the first-stage reaction in the dual-stage reaction leads to an increased B-range abundance. This may be explained as follows: (1) the hydrogenolysis of the species in the B range in the single-stage reaction is dominant, and (2) in the dual-stage reaction, the first-stage catalyst (DM5CQ) may catalyze the hydrogenation of the species in the entire DBE range, with a relatively homogeneous hydrocracking of the hydrogenated molecules over the second-stage catalyst. The C/H ratio after the dual-stage reaction is slightly reduced compared to that after the single-stage reaction. However, the DBE values in all ranges are relatively similar for the feed and products, and the carbon yield of coking should be maintained.

**Figure 2:**
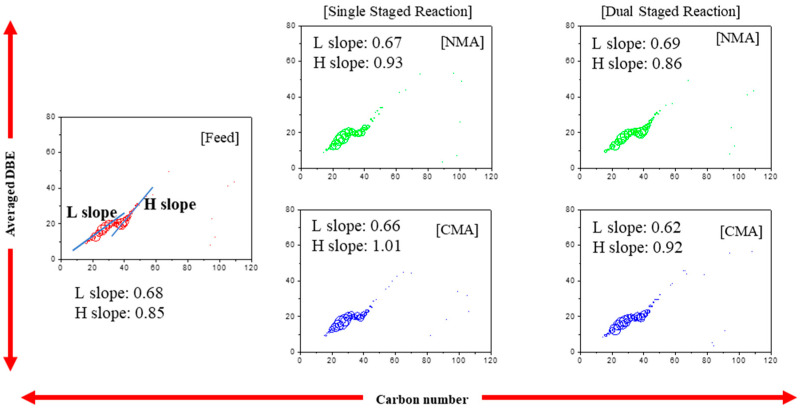
The abundance according to carbon number (C*_c_*H*_h_* species) at the single and dual stages.

Figure 2 shows the abundances as functions of the DBE values and carbon numbers in the feed and products of the single- and dual-stage reactions. For the feed and products of the single- and dual-stage reactions, different types of slopes (Blue line) are obtained for L (light carbons: 12–35 carbon atoms) and H (heavy carbons: 40–68 carbon atoms) molecules. For the single-stage reaction, the values of the L slopes are slightly decreased, which is attributed to the hydrogenation effect, whereas the H slopes after the reaction are higher than that of the feed, which may be attributed to the sites of the removed heteroatoms of the agglomerates of the C_c_H*_h_* heteroatoms.

### 2.4. Subclasses of Nitrogen

Table 4 summarizes the relative abundances of the unitary, binary, and ternary nitrogen heteroatoms in CTP during each stage of CTP hydrotreatment. The sulfur species in CTP may be easily removed, indicating the presence of highly reactive species, whereas the removal of nitrogen species is challenging, which is attributed to the refractory nitrogen species with steric hindrance. Therefore, a detailed investigation of the molecular behavior in nitrogen removal is required to characterize this process. Unitary species are the most abundant in the feed, followed by the binary and then ternary nitrogen species. The total abundance of C*_c_*H*_h_*-N*_n_* decreases during the single- and dual-stage reactions. However, the abundance of C*_c_*H*_h_*-N_4_ species increases, regardless of the reaction type, possibly because of the partial condensation of N*_1_*, N*_2_*, or N*_3_*. The C*_c_*H*_h_*-N*_n_* compounds mainly contain N_1_ species, and these species appear to lead to HDN during the single- and dual-stage reactions. The binary nitrogen species C*_c_*H*_h_*-N*_1_*S*_3_*, -N*_1_*S*_4_*, -N*_2_*S*_2_*, and -N*_3_*S*_1_* are refractory, whereas C*_c_*H*_h_*-N*_1_*S*_1_* represents a relatively reactive species. The more favorable HDO of CTP compared to HDN effectively removes the C*_c_*H*_h_*-N*_n_*O*_o_* species. Research on the development of an effective process for upgrading CTP to high-quality carbon materials should focus on the design of novel catalysts with higher HDN activities.

**Figure 3:**
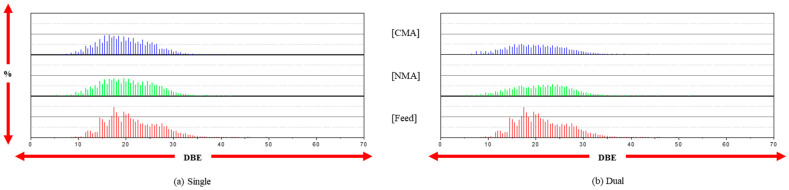
The distribution of DBE (C*_c_*H*_h_*-N*_n_* species) at the single and dual stages.

The distribution of the DBE values follows the C*_c_*H*_h_* trend, as shown in Figure 3. However, the DBE values are significantly reduced in the single- and dual-stage reactions, indicating the effective removal of single heteroatoms, such as the C*_c_*H*_h_*-N*_n_* species. The second-stage reaction, in particular, results in a significant reduction in the amount of C*_c_*H*_h_*-N*_n_* species after mild catalytic hydrogenation in the first stage. However, whether the products of the C*_c_*H*_h_*-N*_n_* species after the second-stage reaction occur in sterically hindered forms is unknown. This may be confirmed by the introduction of a hybrid process, such as catalysis and adsorption.

**Figure 4:**
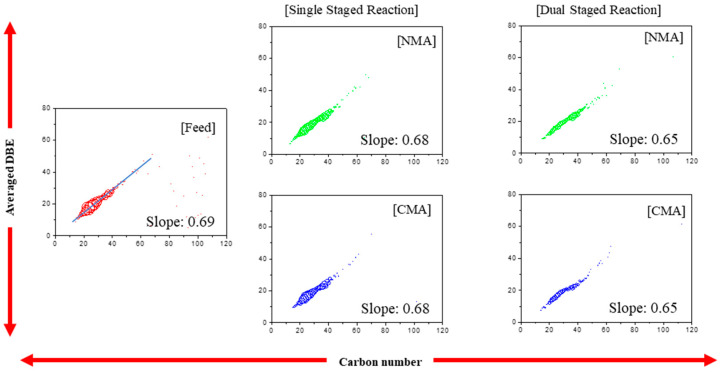
The abundance according to carbon number (C*_c_*H*_h_*-N*_n_* species) at the single and dual stages.

Figure 4 shows the DBE values of C*_c_*H*_h_*-N*_n_* depending on the carbon number. Single slopes are obtained for the feed and products of the single- and dual-stage reactions, in contrast to those of the C*_c_*H*_h_* species. The slopes obtained for all products are low, and nitrogen species are removed via ring opening after hydrogenation. The slopes decrease significantly (0.69 → 0.65) during the dual-stage reaction, indicating accelerated HDN after the first-stage reaction.

## 3. Materials and Methods

### 3.1. Materials

Table 5 summarizes the characteristics of the CTP used in this study. Single- and dual-stage reactions were performed. The commercial catalysts (NMA and CMA) were used as received from JGC Catalysts and Chemicals (JGC C&C, Kawasaki, Japan) in the single-stage reaction. To address the active sites [26,27], the active sites over NiMo and CoMo can be elucidated to symmetric stretching of the Mo–O bond in bridged or two-dimensional polymeric forms of octahedrally coordinated Mo oxide species. Such Mo oxide species have been shown to interact weakly with supports, resulting in higher reducibility and activity in hydrotreating reactions [26]. Moreover, In the Ni(Co)–Mo–S model, Ni(or Co)S is considered the source of promoter atoms and is located in any of the fivefold coordinated sites at the (10$\bar{1}$0) edge planes of MoS_2_. The distribution of MoS slabs over support materials is closely related to the presence of promoters such as Ni and Co, although the sulfidation of calcined catalysts may lead to the redistribution of surface species. The lower number of layered stacks (MoS) leads to a shorter distance between the active sites and the support materials, thereby resulting in improved HDS via effective hydrogenolysis [27]. As-received NMA and CMA catalysts have been characterized by XRF (ZSX Primus IV, Tokyo, Japan), N_2_-sorption isotherm (TriStar II3020, Norcross, GA, USA), TEM (HITACHI HF5000, Tokyo, Japan), Raman (LabRAM HR-800, Tokyo, Japan), and NH_3_-TPD (BELL CAT II, Osaka, Japan) and are presented in the Supplementary Information together with chemical stability data. The dual-stage reaction was performed using a MoO_3_/γ-alumina catalyst (DM5CQ, JGC C&C) in the first reaction to conduct hydrogenation and partial hydrodesulfurization, and NMA and CMA were then introduced in the following (second) reaction to complete the dual reaction. Table 6 lists the characteristics of the basic catalysts.

### 3.2. Hydrotreatment Apparatus

The catalysts were sulfidized prior to hydrotreatment using a gaseous mixture containing 5 vol.% H_2_S in flowing hydrogen at 360 °C for 2 h. CTP hydrotreatment was conducted in a 150 mL autoclave reactor equipped with a sampling port, and the reaction was conducted in one or two stage(s). During a typical experiment, 30 g of feed was hydrotreated in the presence of 3 g of a sulfidized catalyst at 350 °C under 6 MPa hydrogen atmosphere (initial pressure at room temperature). The heating time required to reach the reaction temperature was 0.5 h, and the agitation speed was maintained at 400 rpm to avoid external mass transfer limitations. The reaction time for the one-stage operation was 6 h after reaching the target temperature. In the two-stage operation, first-stage hydrodesulfurization (HDS) was performed for 6 h. This was followed by rapid cooling of the reactor to room temperature and a subsequent second-stage operation with fresh hydrogen. The two-stage operation, wherein the gaseous product was replaced with fresh hydrogen, was conducted to effectively remove heteroatoms over the catalyst.

### 3.3. Bulk Analysiss

The products of the hydrogenation of CTP were used without pretreatment, and an elemental analyzer (EA1110, CE Instruments, Wigan, UK) was used to determine the bulk sulfur and nitrogen contents in CTP.

### 3.4. FT-ICR MS

The 7 T FT-ICR M spectrometer (SolariX 2XR, Bruker, Billerica, MA, USA) used in this study was equipped with a detection system that enabled detection at twice the cyclotron frequency [28]. Nitrogen was used as the drying and nebulization gas; argon was supplied to the collision cell; and the samples were directly injected using a syringe pump (Harvard Apparatus, Holliston, MA, USA). The stock solutions were prepared by diluting the samples in toluene to approximately 0.5 mg/mL, and the APPI source (APPI II, Bruker Billerica, MA, USA) was used in the positive mode. The sample was charged at a flow rate of 300 µL/h. The drying gas temperature and flow rate were maintained at 220 °C and 3.8 L/min, respectively, and the pressure of the nebulizer gas was set to 3.3 bar. The capillary voltage was 3.5 kV. The source was heated to 430 °C, and the mass spectra were acquired between *m*/*z* 180 and 1200. The dataset size was set to 8 M words, and the ion accumulation time was 7.2 s. A total of 200 data points were summed to generate the final spectrum with a signal-to-noise ratio of 4, and the resolving power (M/ΔM_50%_, where ΔM_50%_ is the height of the full width at half-maximum) was approximately 940,000 at *m/z* 400 and 690,000 at *m/z* 700.

The spectra were interpreted using Statistical Tool for Organic Mixture Spectra (STORMS 1.0) software with an automated peak-picking algorithm to obtain rapid and highly reliable results. The elemental formulae were estimated based on the calibrated peak list, and the assignments were based on the *m/z* values, with an error margin of 0.33 ppm. Normal conditions for petroleum data (C*_c_*H*_h_*N*_n_*O*_o_*S*_s_* with c and h unlimited, 0 ≤ *n* ≤ 5, 0 ≤ *o* ≤ 5, and 0 ≤ *s* ≤ 4) were adopted in these calculations. The double-bond equivalent (DBE) value, which represents the number of aromatic rings plus the number of carbon double bonds in each molecular formula, was calculated using the following equation:

DBE = *c* − *h*/2 + *n*/2 + 1 (for elemental formulae C*_c_*H*_h_*N*_n_*O*_o_*S*_s_*)

## 4. Conclusions

Hydrodenitrogenation (HDN) of coal tar pitch was firstly evaluated in the presence of NiMo/γ-alumina (NMA) and CoMo/γ-alumina (CMA). It is generally accepted that hydrodenitrogen catalysts also show the performance of hydrodesulfurization (HDS) during the hydrotreating process. The sulfur removals were recorded at 86.5% and 94.7% in the presence of NMA and CMA, respectively. In contrast, nitrogen removals were obtained much less than expected, showing 26.8% and 29.2% in the presence of NMA and CMA, respectively. With the help of relative abundance analyses using FT-ICP MS equipped with APPI, nitrogen removal performance was further evaluated by analyzing intrinsic nitrogen placed in unitary, binary, and ternary states in coal tar pitch. The results showed that the binary state of C*_c_*H*_h_*-N*_n_*S*_s_* is the most difficult to be removed during the hydrotreating process, even in the presence of HDN catalysts. This study attempted to use a prior-hydrotreating option with a commercial HDS catalyst (DM5CQ), followed by HDN treatment. We named HDS in the presence of DM5CQ as the first-stage treatment and the following HDN in the presence of NMA or CMA as the second-stage treatment. After the first stage, relative abundance data of subclasses were also determined. The sulfur contents in the unitary state (C*_c_*H*_h_*-S_s_) decreased but increased in binary states (C*_c_*H*_h_*-N*_n_*S*_s_*, C*_c_*H*_h_*-O*_o_*S*_s_*) and the ternary state (C*_c_*H*_h_*-N*_n_*O*_o_*S*_s_*). Since the HDS catalyst (DM5CQ) is designed for petroleum heavy fraction, the result might be different. It should be noted that this study focused on the total removal performance of nitrogen removals in the serial hydrotreatments of the first stage followed by the second stage. After dual stages, significant improvement of nitrogen removals was achieved. Both NMA and CMA catalysts showed that a major reduction of nitrogen contents was obtained in the reduction of unitary nitrogen, specifically of N*_1_* and N*_2_* species. In the case of binary nitrogen removal, the NMA catalyst showed a slightly better performance for C*_c_*H*_h_*-O*_o_*N*_n_* molecules than the CMA catalyst; however, almost similar trends were observed for C*_c_*H*_h_*-N*_n_*S*_s_* molecules. In the case of ternary nitrogen, C*_c_*H*_h_*-N*_n_*O*_o_*S*_s_*, no clear differences were observed. Conclusively, the dual stages of hydrotreatments of coal tar pitch resulted in the complete sulfur removal performance and significant improvement of nitrogen removal from the range of 26.8~29.2% to the range of 46.4~48.7%. This implies the fact that nitrogen remnants after dual stages of hydrotreatment are hardly removed because of steric hindrance within the pitch structure.

## Figures and Tables

**Table 1:** Results of elementary analysis.

	Feed	Single-Stage Reaction				Dual-Stage Reaction					
		NMA	Change (%)	CMA	Change (%)	DM5CQ (1st Stage)	Change (%)	NMA (2nd Stage)	Change (%)	CMA (2nd Stage)	Change (%)
C/H	18.8	15.8	−15.9	16.1	−14.3	15.9	−15.4	14.8	−21.3	14.8	−21.3
Total S (wt%)	0.415	0.056	−86.5	0.022	−94.7	0.053	−87.2	-	−100	-	−100
Total N (wt%)	1.33	0.97	−26.8	0.94	−29.2	1.04	−21.8	0.71	−46.4	0.68	−48.7

**Table 2:** Relative abundance of various classes in feed and products during single-stage reaction.

Heteroatoms	Feed	NMA	Change, %	CMA	Change, %
C*_c_*H*_h_*	54.379	67.217	23.6	67.936	24.9
C*_c_*H*_h_*-N*_n_*	20.610	17.072	−17.2	16.381	−20.5
C*_c_*H*_h_*-S*_S_*	1.942	0.336	−82.7	0.179	−90.8
C*_c_*H*_h_*-O*_O_*	10.900	7.650	−29.8	7.673	−29.6
C*_c_*H*_h_*-N*_n_*O*_O_*	6.472	2.774	−57.1	2.885	−55.4
C*_c_*H*_h_*-N*_n_*S*_S_*	2.043	2.200	7.7	2.294	12.3
C*_c_*H*_h_*-O*_O_*S*_S_*	1.500	1.006	−32.9	0.867	−42.2
C*_c_*H*_h_*-N*_n_*O*_O_*S*_S_*	0.974	0.656	−32.6	0.649	−33.4

**Table 3:** Relative abundance of subclasses in feed and products during dual-stage reaction.

Heteroatoms	Feed	DM5CQ (1st Stage)	Change, %	NMA (2nd Stage)	Change, %	CMA (2nd Stage)	Change, %
C*_c_*H*_h_*	54.379	62.567	15.1	74.682	37.3	74.463	36.9
C*_c_*H*_h_*-N*_n_*	20.610	17.553	−14.8	14.111	−31.5	12.053	−41.5
C*_c_*H*_h_*-S*_S_*	1.942	0.514	−73.5	0.118	−93.9	0.138	−92.9
C*_c_*H*_h_*-O*_O_*	10.900	7.293	−33.1	5.284	−51.5	7.309	−32.9
C*_c_*H*_h_*-N*_n_*O*_O_*	6.472	3.879	−40.1	2.313	−64.3	2.709	−58.1
C*_c_*H*_h_*-N*_n_*S*_S_*	2.043	3.861	89.0	1.033	−49.4	1.018	−50.2
C*_c_*H*_h_*-O*_O_*S*_S_*	1.500	2.117	41.1	0.632	−57.9	0.551	−63.3
C*_c_*H*_h_*-N*_n_*O*_O_*S*_S_*	0.974	1.095	12.4	0.606	−37.8	0.549	−43.6

**Table 4:** The subclasses on N species of products in single- and dual-stage reactions.

Heteroatoms	Feed	Single-Stage Reaction		Dual-Stage Reaction		
		NMA	CMA	DM5CQ (1st Stage)	NMA (2nd Stage)	CMA (2nd Stage)
C*_c_*H*_h_*-N*_n_*	20.610	17.072	16.381	17.553	14.111	12.053
N_1_	18.473	16.029	15.262	16.137	13.594	11.471
N_2_	2.053	0.985	1.058	1.358	0.426	0.278
N_3_	0.061	0.024	0.032	0.030	0.055	0.043
N_4_	0.020	0.031	0.026	0.026	0.034	0.026
N_5_	0.003	0.003	0.002	0.002	0.002	0.006
C*_c_*H*_h_*-N*_n_*O*_O_*	6.472	2.774	2.885	3.879	2.313	2.709
N_1_O_1_	4.710	2.136	2.135	2.326	1.808	1.986
N_1_O_2_	0.745	0.141	0.104	0.184	0.180	0.279
N_2_O_1_	0.570	0.062	0.184	0.113	0.035	0.026
N_2_O_2_	0.280	0.283	0.352	1.154	0.188	0.293
N_2_O_3_	0.049	0.013	0.008	0.013	0.012	0.019
C*_c_*H*_h_*-N*_n_*S*_S_*	2.043	2.200	2.294	3.861	1.033	1.018
N_1_S_1_	0.912	0.167	0.200	0.288	0.092	0.130
N_1_S_3_	0.129	0.437	0.330	0.604	0.144	0.181
N_1_S_4_	0.607	1.010	1.228	2.072	0.470	0.405
N_2_S_2_	0.089	0.101	0.097	0.112	0.084	0.096
N_3_S_1_	0.082	0.244	0.206	0.442	0.039	0.034
C*_c_*H*_h_*-N*_n_*O*_O_*S*_S_*	0.974	0.656	0.649	1.095	0.606	0.549
N_1_O_1_S_1_	0.173	0.070	0.072	0.069	0.070	0.070
N_1_O_1_S_2_	0.045	0.046	0.057	0.076	0.059	0.046
N_1_O_1_S_4_	0.120	0.035	0.031	0.169	0.011	0.006
N_1_O_2_S_1_	0.025	0.012	0.010	0.021	0.007	0.007
N_1_O_3_S_1_	0.029	0.025	0.030	0.039	0.013	0.005

**Table 5:** Basic properties of coal tar pitch (as-received).

	Softening Point (°C)	Toluene Insoluble (%)	Quinoline Insoluble (%)	Beta-Resin (%)	Ash (%)	Viscosity at 0.84 s^−1^, cP		
						170 °C	190 °C	230 °C
CTP, as-received	110–120	25.4	4.9	20.5	0.24	1860	460	54.4

**Table 6:** Basic properties of as-received catalysts.

Characteristics	Unit	Catalysts (Single/Dual Stage)		
		1st Stage	2nd Stage	
		DM5CQ	NMA	CMA
Bulk density	g/cc	0.43	0.61	0.60
Surface area	m^2^/g	178	232	228
Average pore diameter	nm	17.4	9.8	10.2
Active metal		Mo	Ni-Mo	Co-Mo
Metal contents				
Mo	wt. %	2.7	7.6	7.7
Ni	wt. %	-	2.5	-
Co	wt. %	-	-	2.6

## Data Availability

Not applicable.

## Supplementary Materials

The supporting information can be downloaded at: https://www.mdpi.com/article/10.3390/ijms241411793/s1.
