# Supplementary material for: Molecular Characteristics of Catalytic Nitrogen Removal from Coal Tar Pitch over γ-Alumina-Supported NiMo and CoMo Catalysts
Source: Int J Mol Sci. 2023 Jul 22;24(14):11793. doi: 10.3390/ijms241411793 (PMC10380854; doi:10.3390/ijms241411793)

Supplementary Information

1. Stability Data of Catalysts

	Feed	Single Staged Reaction									
		NMA					CMA				
		1st cycle	2nd cycle	3rd cycle	4th cycle	5th cycle	1st cycle	2nd cycle	3rd cycle	4th cycle	5th cycle
**C/H**	18.8	15.8	15.8	15.9	15.8	15.8	16.1	16.0	16.1	16.2	16.1
**Total S (wt%)**	0.415	0.056	0.056	0.055	0.057	0.057	0.022	0.020	0.022	0.021	0.021
**Total N (wt%)**	1.33	0.97	0.99	0.99	0.98	0.97	0.94	0.94	0.93	0.93	0.94

*** All Cycle tests have been carried out after enough Soxhlet of catalysts**

2. Characterization of Catalysts

XRF Results on NMA and CMA

(After Sulfidation) [Unit: %]

		NMA	CMA
Supports	Al_2O_3	63.239 (51.172)	53.116 (54.682)
	SiO_2	-	-
Active Metals	MoO_3	19.497 (17.732)	8.625 (12.168)
	NiO	4.594 (4.111)	-
	Co_2O_3	-	2.776 (4.031)
Additives	P_2O_5	2.610 (2.660)	1.918 (2.779)
	Na_2O	-	26.371 (-)
	SO_3	10.060 (24.325)	7.194 (26.278)
	CaO	-	-

N_2-Sorption Isotherm on NMA and CMA

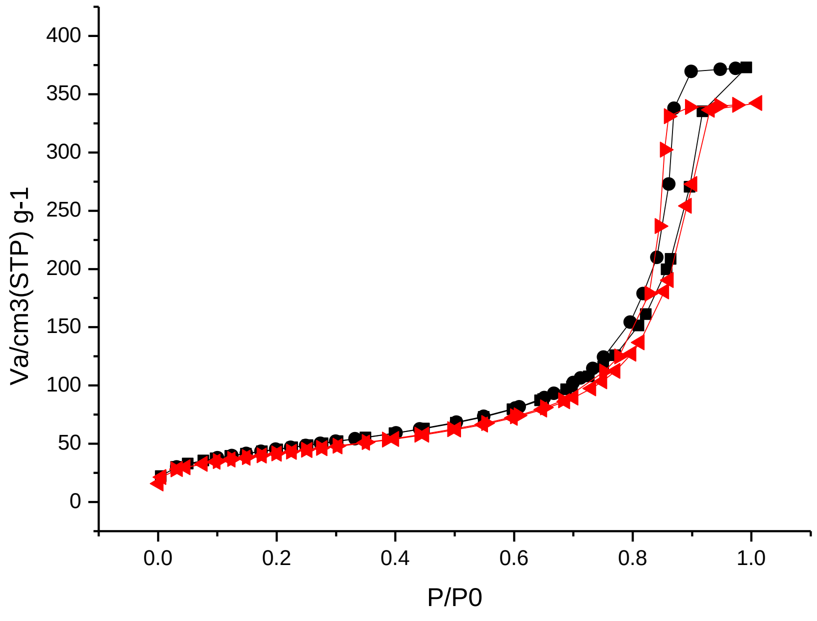

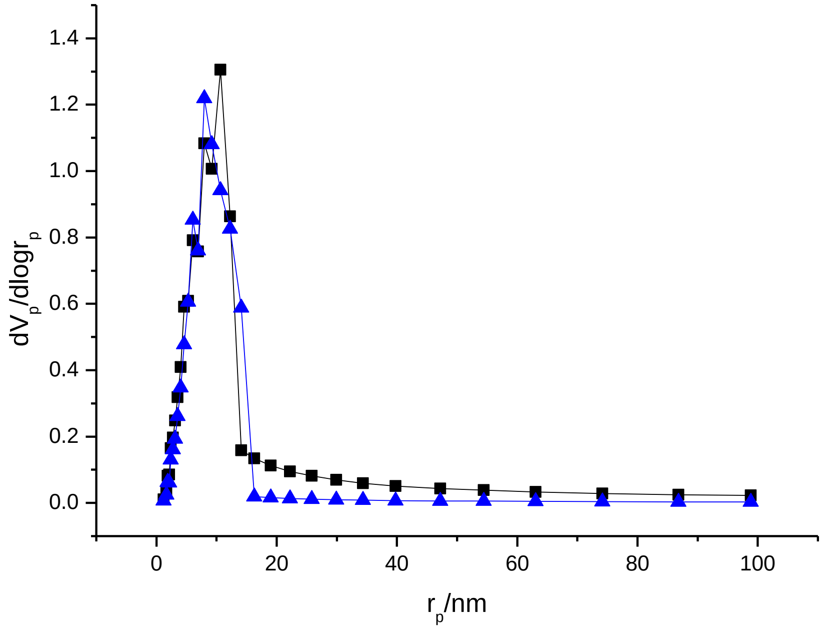

	NMA	CMA
BET Surface Area (m^2/g)	149	163
Ave. Pore Size (nm)	14.1	14.1
Total Pore Volume (m^3/g)	0.53	0.58
Ave. Pore Volume (m^3/g)	0.53	0.57

Raman Spectra on NMA and CMA

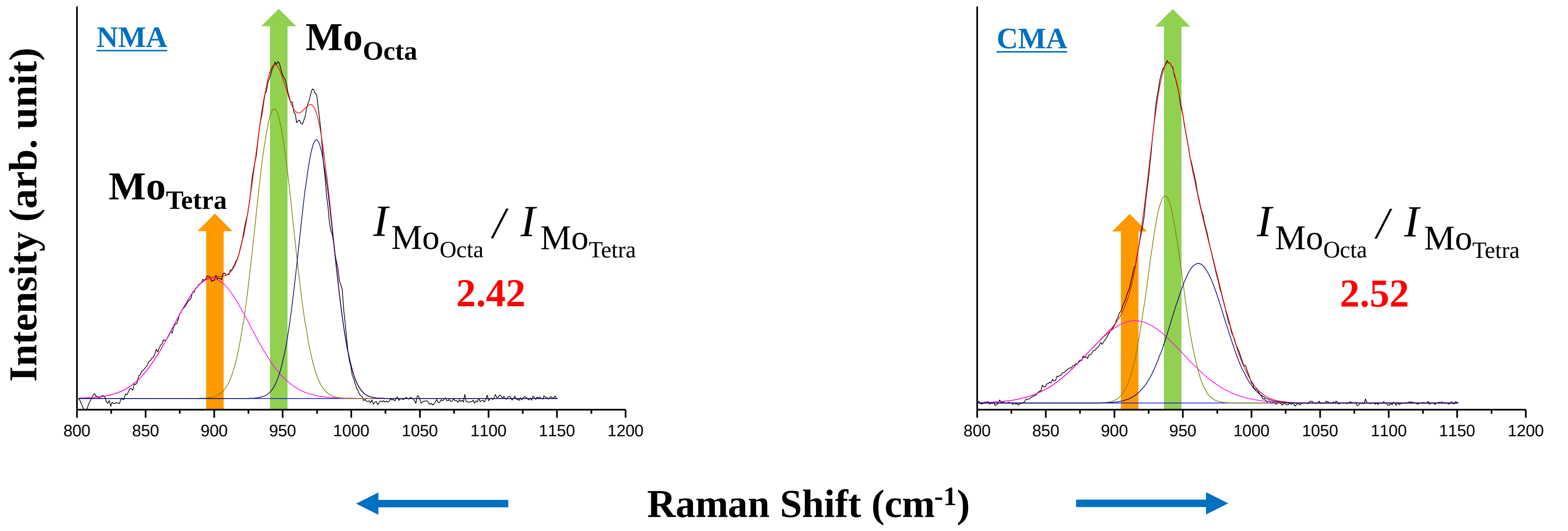

Summary of Layer Number on NMA and CMA (From TEM Images)

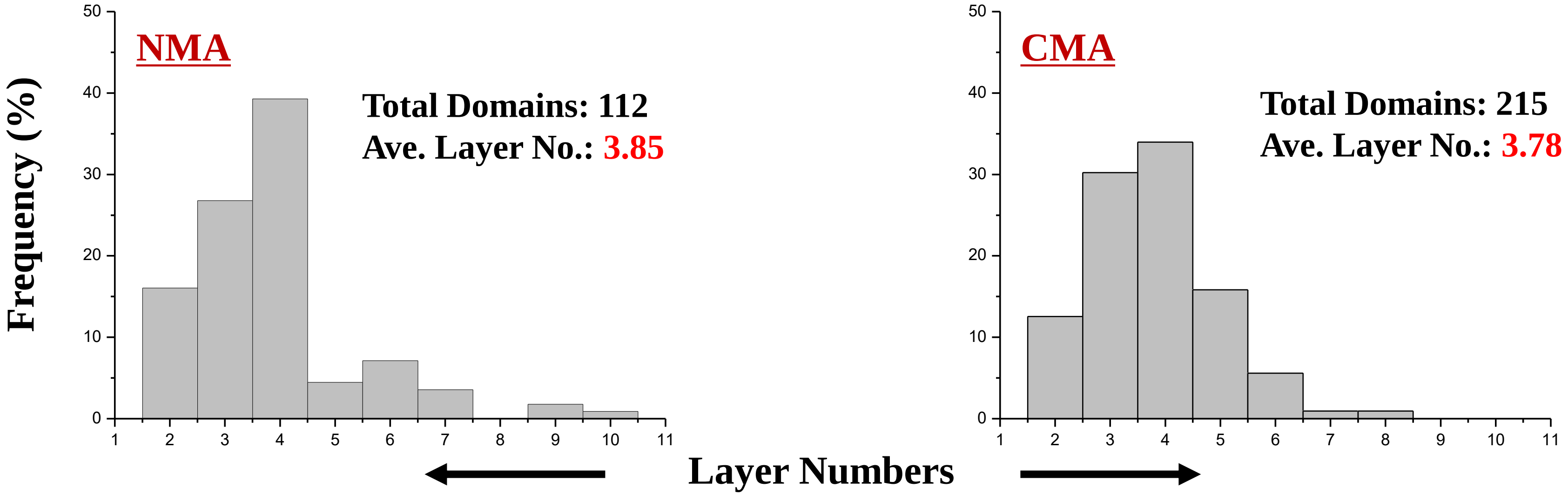

Layer No.	NMA		CMA	
	Counts	Frequency (%)	Counts	Frequency (%)
2	18	16.1	27	12.6
3	30	26.8	65	30.2
4	44	39.3	73	34.0
5	5	4.5	34	15.8
6	8	7.1	12	5.6
7	4	3.6	2	0.9
8	0	0.0	2	0.9
9	2	1.8	0	0.0
10	1	0.9	0	0.0
Total	215	100	215	100.0
Ave. Layers	3.85		3.78	

NH_3-TPD on NMA and CMA

[Unit: mmol/g]

	Weak Acidity<br>-490°C	Medium Acidity<br>490-770°C	Strong Acidity<br>770°C-	Total Acidity
NMA	0.89	0.17	0.01	1.07
CMA	1.01	0.14	0.01	1.16

TEM Images and Layer Number (NMA)

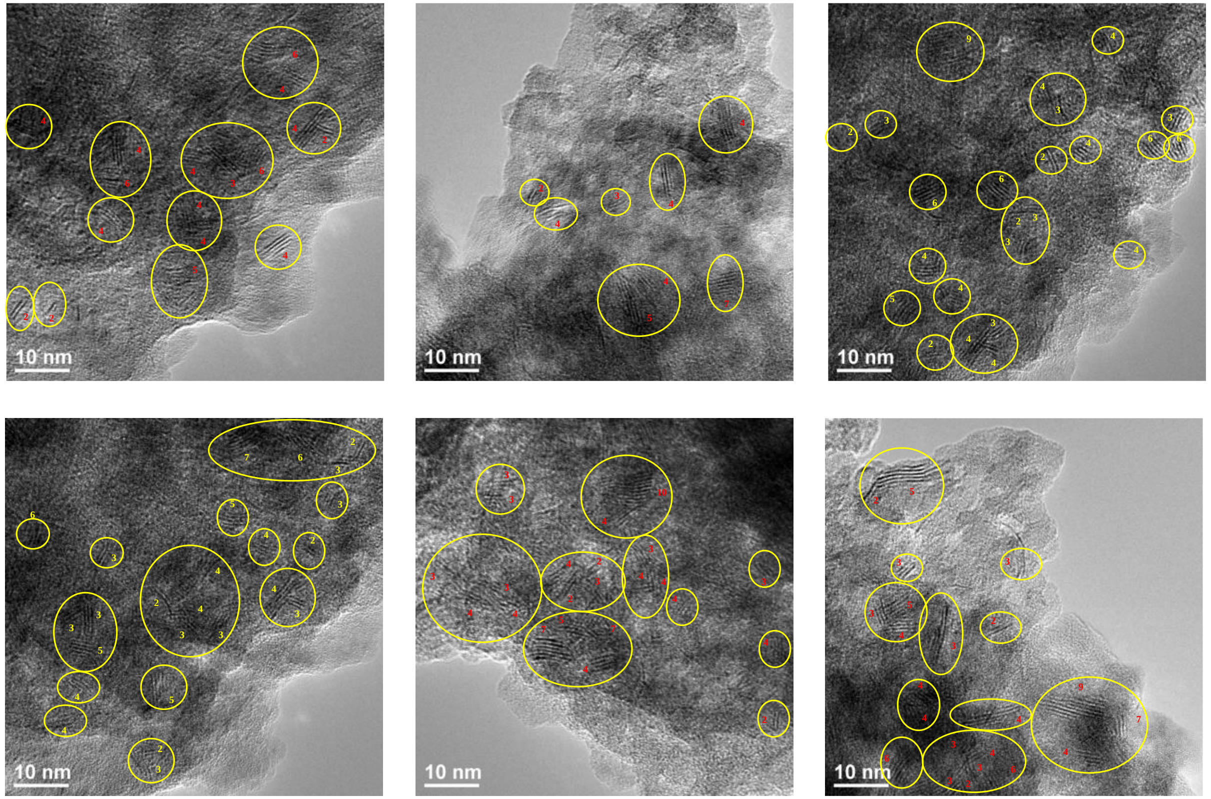

TEM Images and Layer Number (CMA)

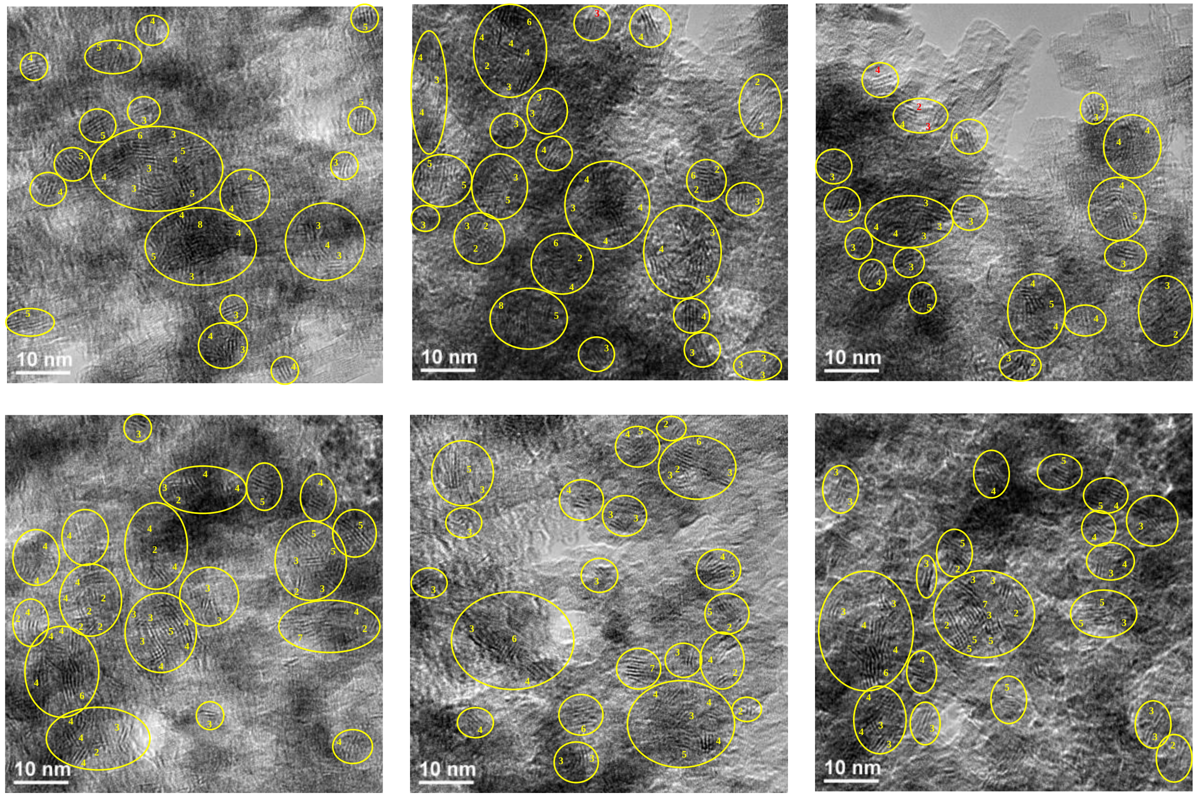

Supplement: Supplementary file 1 [file ijms-24-11793-s001.zip › ijms-2465804-supplementary.pdf]
